# Seasonal pollution and surface characteristics of microplastics in surface water in the Wanzhou section of the Three Gorges Reservoir, China

**DOI:** 10.1007/s11356-023-27185-w

**Published:** 2023-04-29

**Authors:** Ying He, Qian Huang, Qilong Wang, Mingfeng Tang, Xiaoyu Lu, Fei Cheng, Guosheng Xiao

**Affiliations:** 1 College of Biology and Food Engineering, Chongqing Three Gorges University, Wanzhou, Chongqing, China; 2 Key Laboratory of Water Environment Evolution and Pollution Control in Three Gorges Reservoir, Chongqing Three Gorges University, Wanzhou, Chongqing, China; 3 Engineering Technology Research Center of Characteristic Biological Resources in Northeast Chongqing, Chongqing Three Gorges University, Wanzhou, Chongqing, China

**Keywords:** Microplastic, Three Gorges Reservoir, Surface water, Seasonal pollution

## Abstract

The pollution of freshwater environments with microplastics (MPs) has attracted increasing attention owing to their threats to aquatic ecosystems and human health. Here, we sampled and analyzed MPs from mainstream, tributary, and backwater areas in the Wanzhou section of the Three Gorges Reservoir (TGR) in impoundment and flood periods. Microplastic pollution was the most severe in the backwater areas. The average abundance of MPs reached the highest value in the flood period (5.27±3.47×10^7^ items km^−2^), which was 3–5 times that in the impoundment period. In the 0.3–5 mm size class, the 1–5 mm fraction was the most abundant, accounting for more than 81% in the flood period and 68% of the total MP particle abundance in the impoundment period in the mainstream and backwater areas. However, 0.3–1 mm MPs contributed more than 50% in the tributaries during the impoundment period. Polystyrene, polypropylene, and polyethylene MPs were detected in foam, fragment, sheet, and line-shaped MP particles. White, opaque, foamed polystyrene MPs contributed 32–81% to total MP particle abundance in the watershed. Microplastic particle surfaces showed signs of damage and oxidation, and ten different elements were found. Oxygen was clustered on the surface of foam and fragment MPs. Microplastic pollution was severe in the Wanzhou watershed. Especially in the backwater areas, oxidized MPs of variable shapes derived mainly from surface runoff in the flood period and sewage discharge in the impoundment period were abundant. The results of this study contribute to understanding seasonal pollution patterns and surface characteristics of MPs in the TGR and similar watersheds.

## Introduction

Plastics have been widely used to substitute other materials all over the world. China has become a major plastic manufacturing country. According to the statistics of China’s plastic processing industry, the national output of plastic products in China has reached 76.03 million tons in 2020, of which 2.50 million tons was produced in Chongqing municipality (Ma and Jang 2021). While plastic products are convenient for human life, they pose a serious environmental pollution risk. Used plastic products break down or degrade into microplastics (MPs, <5mm) that enter aquatic environments continuously and are ingested by organisms in large quantities (Wagner and Lambert 2017; Duis and Coors 2016). As a result, MPs have become a new global environmental pollutant (Browne et al. 2007; Li et al. 2016). They float and are transported in water or sink to the sediments long after entering the water body. Moreover, MPs can combine with certain chemicals (Crawford and Quinn 2017), form biofilms with microorganisms (Zettler et al. 2013; Jiang et al. 2018; Miao et al. 2019), and become carriers of pathogens in water (Viršek et al. 2017; Bowley et al. 2021), causing lasting and cumulative pollution to the environment and potential threats to organisms (Bonfanti et al. 2021).

The impacts of MP pollution on the marine environment have received much attention globally, mostly in the past 20 years. Since MP particles were firstly reported in freshwaters in 2011 (Zbyszewski and Corcoran 2011), MP pollution in freshwater environments has been increasingly studied. MPs in freshwater environments are divided into primary MPs originating from industrial products in cosmetics and industrial raw materials and secondary microplastics formed by the physical, chemical, and biological decomposition of large plastic waste (Carr et al. 2016; Liu et al. 2018; Chen et al. 2020a; Yang et al. 2021). MPs in inland waters are mainly derived from airborne sources (urban and industrial dust), productive activities (fishery, agriculture, and aquaculture activities), wastewater (sewage discharge, wastewater treatment plants), and terrestrial runoff from agricultural and urban areas (Zhao et al. 2020; Ding et al. 2021). In China, MPs are widespread in freshwater environments, including rivers (e.g., Weihe and Zhangjiang rivers) (Ding et al. 2019; Pan et al. 2020), lakes (Dongting, Honghu, Poyang, and Changsha lakes) (Wang et al. 2018; Yin et al. 2019; Yuan et al. 2019), reservoirs (the TGR) (Zhang et al. 2015; Zhang et al. 2017; Di and Wang 2018), and estuaries (Pearl River and Yangtze River estuaries) (Zhao et al. 2014; Yan et al. 2019; Zhao et al. 2019). Reports have shown that MP pollution of freshwater environments is severe in China, but the pollution characteristics of MPs in freshwater are still little studied.

The Three Gorges Reservoir (TGR), located in the upper reaches of the Yangtze River, is the largest freshwater reservoir in China and plays an important role in the socio-economic development of the adjacent regions by optimizing water resources (Huang et al. 2020). However, MPs from industrial and household sewage, wastewater treatment plant discharges, garbage dumping, and agricultural pollution enter the TGR through point and non-point sources (Chang et al. 2010; Di and Wang 2018; Ding et al. 2021). As a result of the impoundment of the Three Gorges Dam (TGD), the reservoir can be an important environmental compartment for MP pollution (Zhang et al. 2019), retaining and accumulating MP (Zhang et al. 2015). Microplastic pollution has been reported in the mainstream near the TGD (Zhang et al. 2015), but detailed studies are missing. In recent years, severe MP pollution has been reported for surface waters, sediments, the hydrofluctuation belt in the mainstream, and tributaries of the TGR (Zhang et al. 2015; Zhang et al. 2017; Di and Wang 2018; Zhang et al. 2019; Zhang et al. 2022). Previous studies on MPs in the TGR have focused mainly on unitary sampling and pollution analysis in some periods. In our study, we analyzed seasonal pollution characteristics of MPs in the mainstream, tributaries, and backwater areas in the Wanzhou section of the TGR, as well as detailed surface characteristics of MP particles. Our study contributes to a better understanding of seasonal pollution patterns and surface characteristics of MPs in the Wanzhou section of the TGR and similar watersheds.

## Materials and methods

### Study area

**Fig. 1:**
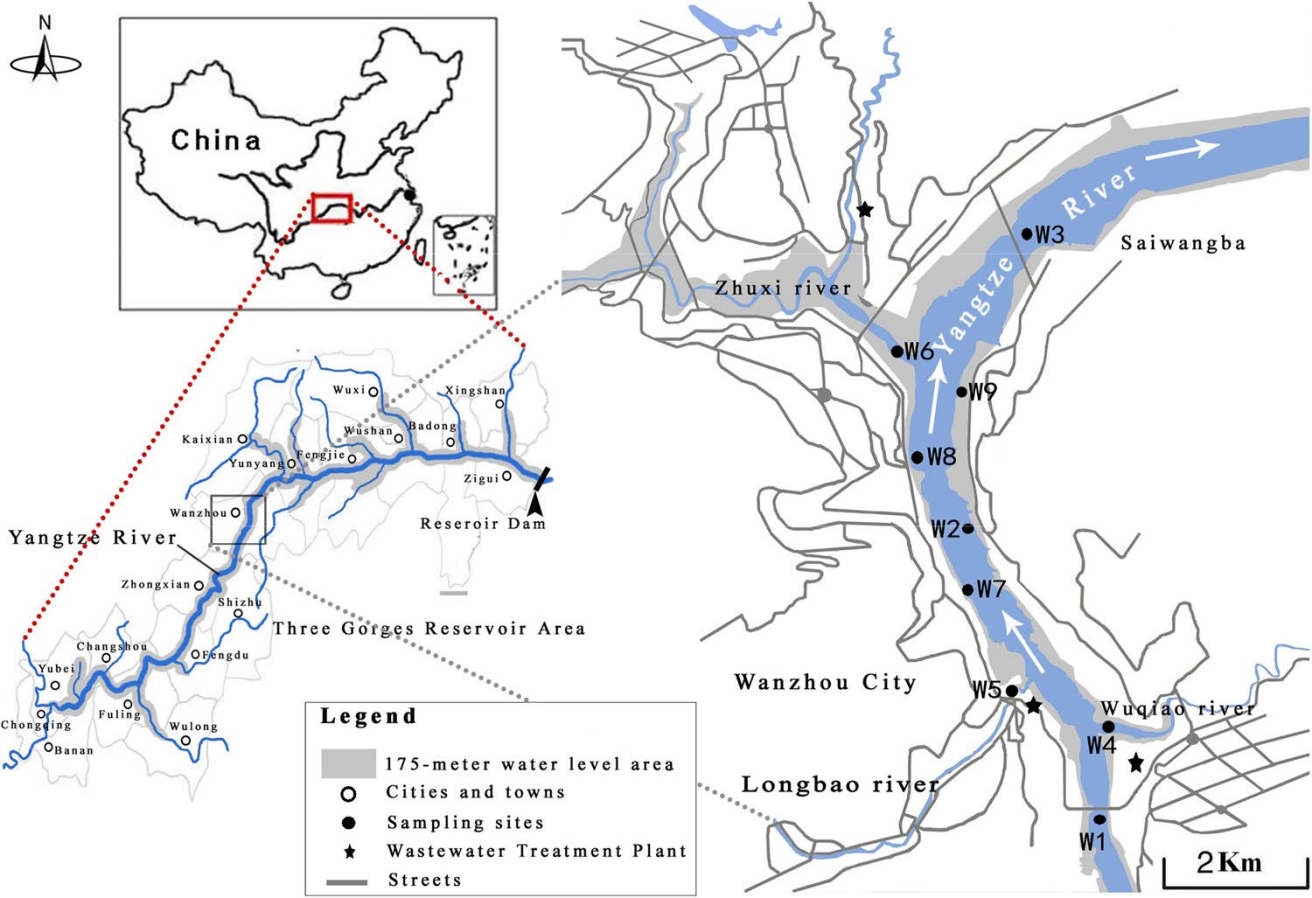
Geographic information of sampling sites in the mainstream (W1, W2, and W3), tributaries (W4, W5, and W6), and backwater areas (W7, W8, and W9) in the Wanzhou section of the Three Gorges Reservoir

After the construction of the Three Gorges Dam in 2009, the TGR became the world’s largest river-type reservoir with the largest and the longest seasonal water-level fluctuations in the Yangtze River. The water level of the TGR ranges between 145 m a.s.l. in the flood period (June to September with heavy rainfall in the upstream section, high flow velocity, and water discharged from the reservoir) and 175 m in the impoundment period (October to May with low rainfall, low flow velocity, and the water retained by a cofferdam in the drought season) (Gao et al. 2015). Wanzhou is a district of Chongqing (China) at the Yangtze River with at about 800,000 population. The Wanzhou section is in the middle of the TGR (Fig. 1). Microplastic samples were taken from surface water at nine sites in the Wanzhou section of the TGR (30°24′25″–31°14′58″ N, 107°55′22″–108°53′25″ E) (Fig. 1). Three sampling sites were in the mainstream of the Yangtze River, i.e., the upper reaches (W1), the middle reaches (W2), and the lower reaches (W3) of Wanzhou city. Another three sites were in the tributaries of the Yangtze River, i.e., Wuqiao River (W4), Longbao River (W5), and Zhuxi River (W6). Finally, the last three sampling sites were in backwater areas of the mainstream of the Yangtze River (W7, W8, and W9).

### Sample collection

Samples (floating debris) were taken once per site at a water level of 175 m during the impounding period on December 1, 2020, and 148 m during the flood period on July 30, 2021, using a quadrate-tapered trawl and following a previously published method with slight modifications (Zhang et al. 2015). Briefly, a self-made trawl with a rectangular opening 30 cm high, 40 cm wide, and 150 cm long made from a 300 μm mesh nylon net (He et al. 2021) was fixed at the side of a boat and dragged along the flow direction in the mainstream and backwater areas and perpendicular to the flow direction in the tributaries in 15–20 cm surface water depth for 20 minutes to collect MPs. The trawling speed was measured using an acoustic Doppler current profiler (FlowQuest 600-AFA-BC, LinkQuest Inc., USA). Trawling area per sample was calculated by multiplying trawling distance with trawl width, while trawling distance equals trawling speed multiplied by trawling time. The collected samples were transferred into a closed container with an ice bag, brought back to the laboratory directly after sampling, transferred to a glass bottle with 5% methyl aldehyde, and stored in a refrigerator at 4 °C before analysis (Zhang et al. 2015; Zhang et al. 2017).

### Sample separation and identification

Samples were digested with 30% H_2_O_2_ at room temperature for 24 h to remove excess impurities (Liebezeit and Dubaish 2012; Scheurer and Bigalke 2018). Then, the MPs were selected and categorized using the standardized size and color sorting (SCS) system (Crawford and Quinn 2017). The color, size, and shape of each MP in each sample were recorded. Microplastic abundance in the water samples is equal to the number of MPs (items) divided by trawling area (km^−2^). Briefly, the digested samples were diluted with distilled water and then sorted by rinsing through 5 mm and 1 mm stainless steel sieves into 3 size classes: >5 mm, 1–5 mm, and <1 mm. The liquid passed through the 1 mm sieve was collected. Particles retained on the 1 mm sieve were picked out using a stainless-steel tweezers, and suspected MPs were inspected visually by the naked eye or under a stereomicroscope. The liquid passing the 1 mm sieves was transferred into a 1 L beaker, then covered with tin platinum paper, and left to stand overnight. The liquid was filtered through a 1.6 μm microporous filter membrane under a vacuum. The particles on the filter membrane were retrieved after suction filtration and identified under a stereomicroscope. The MP separation process was carried out on a closed, ultra-clean workbench to prevent external pollution.

50 to 100 suspected MPs randomly selected from each site by the naked eye and stereomicroscopy were identified using Attenuated Total Reflectance-Fourier Transform Infrared Spectroscopy (ATR-FTIR; Nicolet iS5, Thermo Fisher Scientific Inc.) (Hidalgo-Ruz et al. 2012; Zhang et al. 2015). The FTIR spectrum was recorded from 600 to 4000 cm^−1^. The chemical composition of the MPs was determined by comparing the obtained FTIR spectra with the reference spectra in the database on the instrument using the OMNIC Specta software (Thermo Fisher Scientific Inc.). The MPs were confirmed if they had a high similarity (at least 90%) with the types of references in the database. The identified MPs were picked out and coated with a thin gold film and then were analyzed by Energy Dispersive X-Ray Spectroscopy (EDS, Oxford Xplore 30) in conjunction with Scanning Electron Microscopy (SEM; Zeiss Sigma 300, Carl Zeiss AG, Germany) to observe their surface morphological structures and elemental compositions.

### Data analysis

We performed statistical analysis and data processing using SPSS Statistics 26.0 and drew figures using GraphPad Prism 8.0 software. Data were presented as the mean ± standard derivation (SD). Differences among site types and seasons were analyzed by analysis of variance (ANOVA). We considered *P* < 0.05 as significant and *P* < 0.01 as highly significant.

## Results

### Abundance of microplastics

**Fig. 2:**
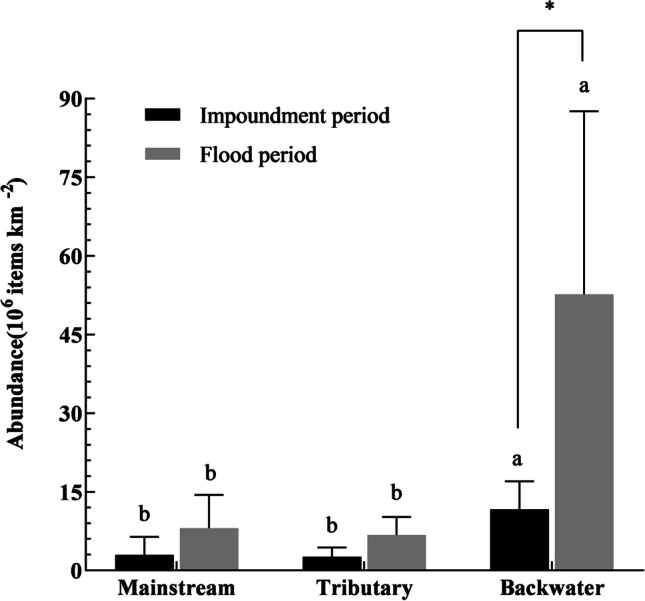
Average abundances of microplastics. Bars represent means ± SD, and different lowercase letters (a and b) and stars (*) indicate significant differences (*P <* 0.05) among three site types in the same period and between the impoundment and flood periods among the same site type, respectively

A large number of MP particles were detected in the mainstream, tributaries, and backwater areas. The MP pollution was the most serious in backwater areas with higher abundances at all sites in both flood and impoundment periods (Table 1). The average abundance in the backwater area was significantly higher than that in the mainstream and tributary (*P* < 0.05) (Fig. 2). The MP pollution was more severe in the flood period with higher abundances at other sites except site W3 than in the impoundment period (Table 1), especially in the backwater area with average abundances of 52.70±34.69 × 10^6^ items km^−2^ in the flood period and 11.60±5.17 × 10^6^ items km^−2^ in the impoundment period (*P* < 0.05, Fig. 2).

**Table 1:** Trawling time, speed, distance and area, and microplastic (MP) abundance for the sampling sites in the flood and impoundment periods in the Wanzhou section of the TGR

Site type	Sample site	Trawling time (min)	Trawling speed (m s^−1^)	Trawling distance (m)	Trawling area (10^−4^ km^2^)	MP numbers (items)	MP abundance (10^6^ items km^−2^)	Abundance of two-size MPs (10^6^ items km^−2^)		Abundance of four-shape MPs (10^6^ items km^−2^)				Average abundance (10^6^ items km^−2^)
								300μm-1mm	1mm-5mm	Foam	Fragment	Sheet	Line	
Impoundment period (December 1, 2020)														
Mainstream	W1	20	0.58	696	2.78	134	0.48	0.40	0.09	0.05	0.11	0.05	0.27	2.95±3.44
	W2	20	0.63	756	3.02	2078	6.87	1.44	5.43	4.43	1.59	0.50	0.35	
	W3	20	0.69	828	3.31	492	1.49	0.98	0.50	0.44	0.38	0.14	0.53	
Tributary	W4	20	0.61	732	2.93	361	1.23	0.72	0.52	0.29	0.30	0.15	0.50	2.70±1.99
	W5	20	0.56	672	2.69	1335	4.97	2.59	2.38	1.54	1.65	1.17	0.61	
	W6	20	0.63	756	3.02	577	1.91	1.32	0.59	0.73	0.85	0.22	0.11	
Backwater	W7	20	0.48	576	2.30	2082	9.04	5.10	3.94	2.04	3.16	2.04	1.79	11.60±5.17
	W8	20	0.57	684	2.74	4802	17.55	2.74	14.81	13.06	3.12	1.13	0.24	
	W9	20	0.54	648	2.59	2127	8.21	2.25	5.96	5.11	2.13	0.60	0.37	
Flood period (July 30, 2021)														
Mainstream	W1	20	0.51	612	2.45	1977	8.08	0.52	7.56	7.23	0.44	0.26	0.14	8.15±6.38
	W2	20	0.87	1044	4.18	6080	14.56	0.99	13.57	12.39	1.25	0.67	0.25	
	W3	20	0.8	960	3.84	692	1.80	1.38	0.42	0.17	0.58	0.44	0.61	
Tributary	W4	20	0.86	732	2.93	1384	4.73	2.66	2.07	1.70	1.70	0.93	0.40	6.24±1.86
	W5	20	0.61	588	2.35	1955	8.31	2.03	6.28	6.23	1.11	0.55	0.42	
	W6	20	0.47	1032	4.13	2349	5.69	0.40	5.29	4.95	0.48	0.13	0.12	
Backwater	W7	20	0.86	1032	4.13	10829	26.23	4.91	21.33	19.20	3.28	2.71	1.05	52.70±34.69
	W8	20	0.46	552	2.21	20308	91.97	17.28	74.69	77.52	6.30	3.44	4.71	
	W9	20	0.72	864	3.46	13783	39.88	6.64	33.24	30.89	3.20	3.04	2.75

### Particle size of microplastics

**Fig. 3:**
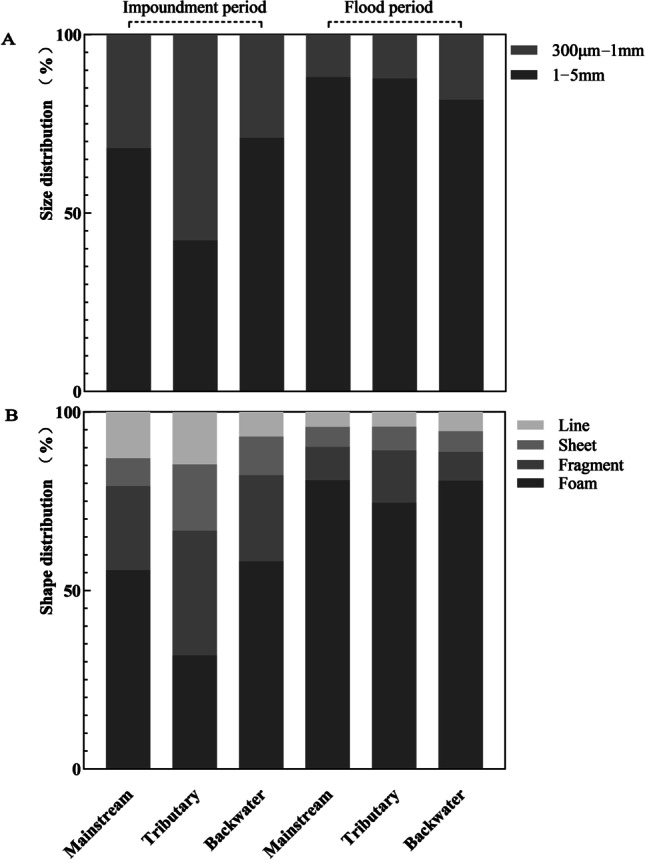
Size (**A**) and shape (**B**) distributions of microplastics in the mainstream, tributary, and backwater area in the impoundment and flood periods

In the flood period, 1–5 mm MPs were the main size fraction found in surface water of the mainstream, tributaries, and backwater areas of the Wanzhou section, accounting for more than 81% of the 0.3–5 mm particle size range (Fig. 3A). In the impoundment period, the proportion of 0.3–1 mm MPs was higher than in the flood period, but in the mainstream and the backwater areas of the Wanzhou section, the pollution of 1–5 mm MPs was still dominant, accounting for more than 68% of the 0.3–5 mm particle size range. However, in the tributaries, the proportion of 0.3–1 mm MPs was higher than that of 1–5 mm MPs, accounting for more than 50% (Fig. 3A).

The MP abundance at the sampling points ranged between 0.48 and 91.97×10^6^ items km^−2^ in the different periods (Table 1). The MP pollution of backwater areas (W7–9) was significantly higher than that of other areas both in the flood and impoundment periods, and 1–5 mm MP particles dominated all sampling points in backwater areas. In the impoundment period, MP pollution was the lowest in the upstream reaches (W1) with a dominance of 0.3–1 mm particles, followed by the downstream reaches (W3). In the flood period, MP pollution was the lowest in the downstream reaches (W3), mainly with 1–5 mm particles, followed by Wuqiao River (W4).

### Shape and color of microplastics

**Fig. 4:**
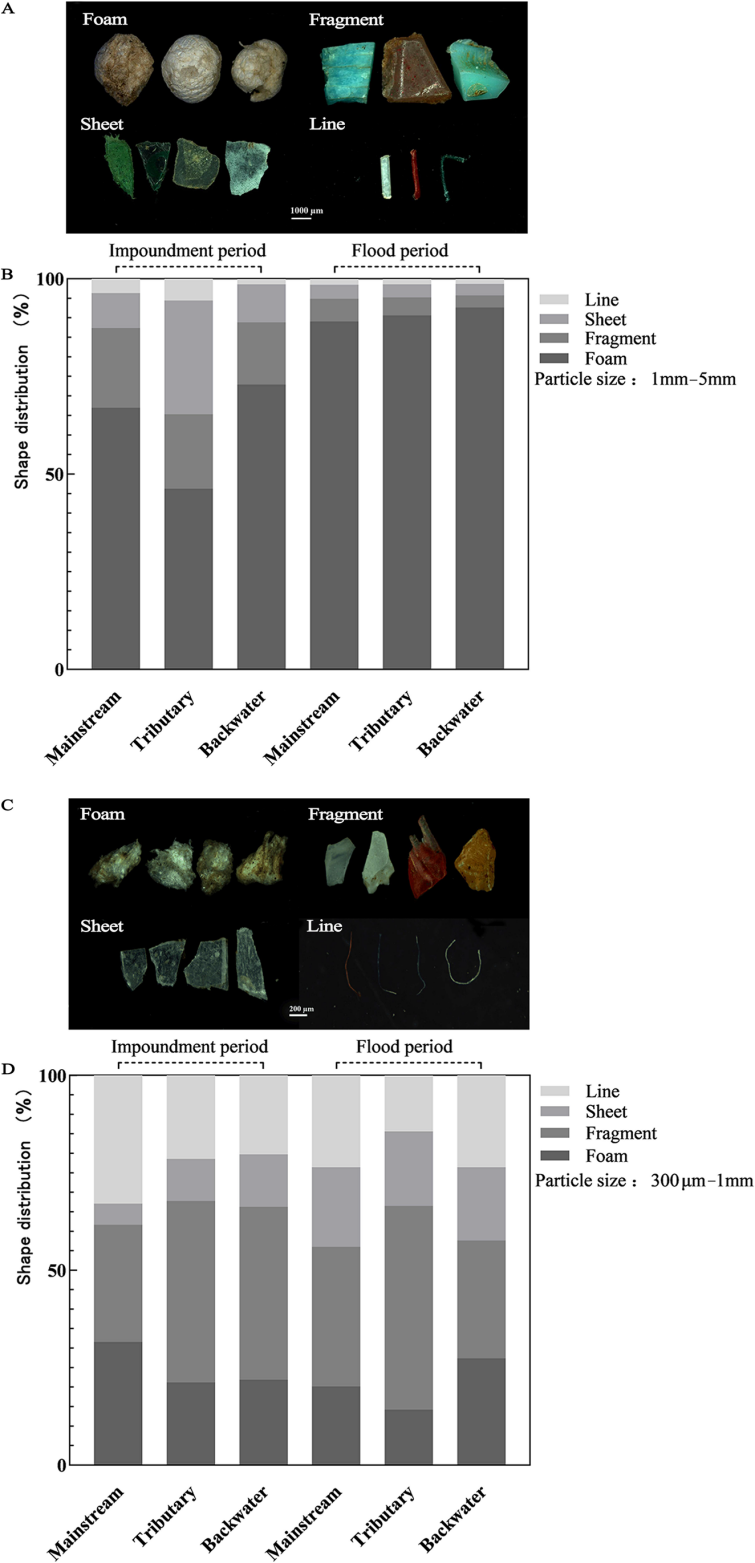
Color and shape distributions of 1–5mm (**A, B**) and 0.3–1mm (**C, D**) microplastics in the mainstream, tributary, and backwater area in the impoundment and flood periods

There were at least four shapes of MPs within the 0.3–5 mm fraction, i.e., foam, fragment, sheet, and line-shaped particles. In the flood period, foam MP particles were relatively abundant in the mainstream, tributary, and backwater areas, reaching 75–81% of total MP particle abundance, followed by fragment, sheet, and line-shaped particles (Fig. 3B). In the impoundment period, foam MPs accounted for over 50% in mainstream and backwater areas. However, in the tributaries, fragment MPs were most frequent, accounting for 35%, followed by foam MPs with 32% (Fig. 3B). In the same size range, the color and shape distribution of MPs were different (Fig. 4). In the mainstream, tributary, and backwater areas, 1–5 mm foam MPs were the most abundant, with more than 46% in the impoundment period and more than 89% in the flood period (Fig. 4B), while fragment MPs were the most abundant in the 0.3–1 mm class. However, the difference in the proportions of various shapes was small (Fig. 4D).

White, red, blue, pink, black, yellow, and green MP particles were encountered, and foam MPs were white and opaque, while the most fragment, sheet, and line-shaped MPs were transparent (Fig. 4A, C).

### Microplastic chemical composition

At least three MP material types were identified by Attenuated Total Reflectance-Fourier Transform Infrared Spectroscopy from MPs in the Wanzhou section of the TGR during the impoundment and flood periods, i.e., polystyrene (PS), polyethylene (PE), and polypropylene (PP). Of all identified MPs, PS was the most common type accounted for 47.3%, followed by 30.4% of PP and 22.3 % of PE. PS MP particles were mainly foam-shaped, PE particles were fragment, sheet, and line-shaped, and PP particles were fragment-shaped. There were other peaks than the characteristic peaks of PP, PE, and PS in the FTIR spectra of MPs, such as peaks of C=O and C-O, but other specific components could be retrieved due to the weak infrared absorption peak.

### Surface microscopic characteristics

**Fig. 5:**
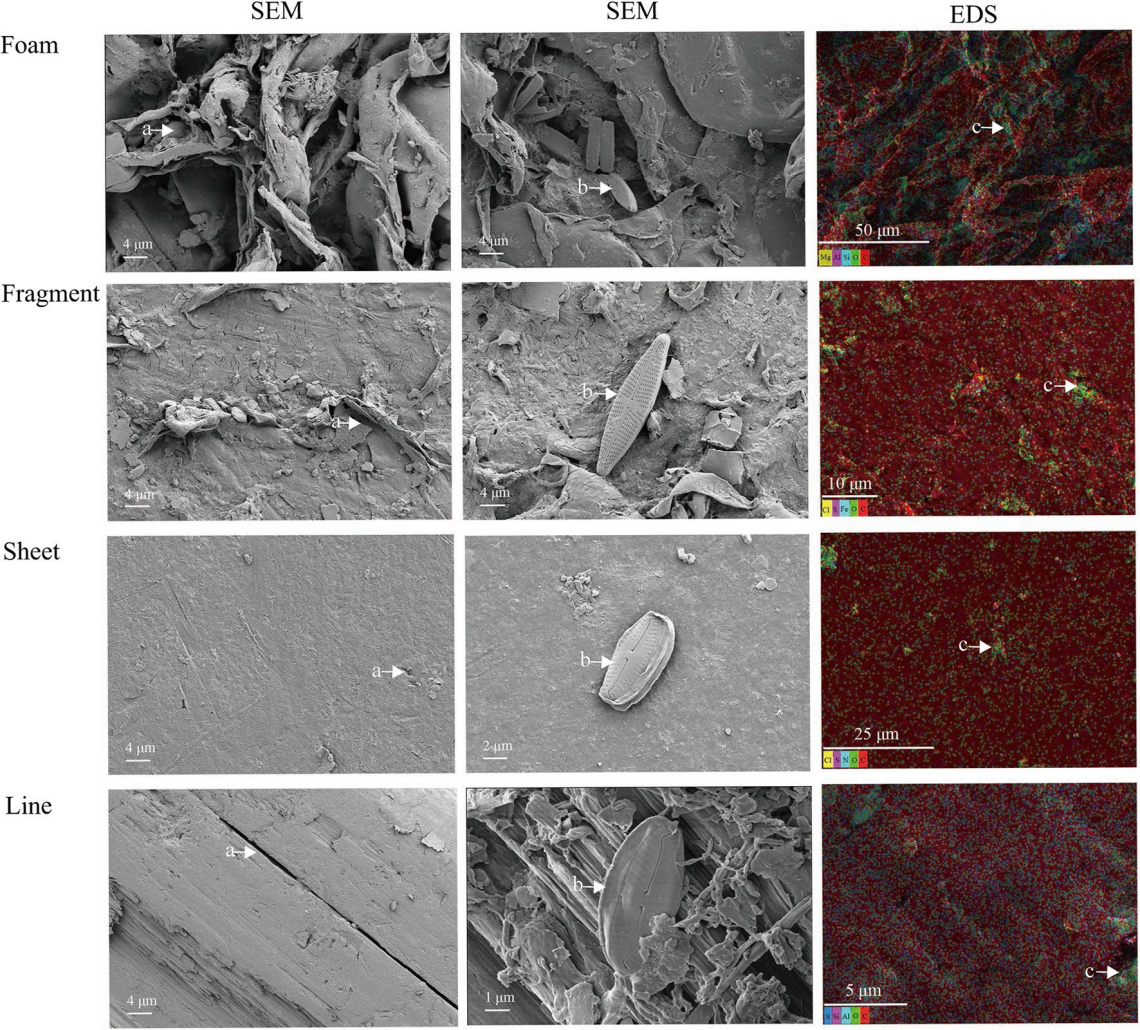
SEM image of foam, fragment, sheet, and line-shaped microplastics and elemental analysis using SEM-EDS. Mechanical damage (a arrows), adherent micro-organisms (b arrows), and clustered oxygen (c arrows)

The SEM surface micromorphology of MP particles differed between shapes across the impoundment and flood periods. The surfaces of foam, fragment, sheet, and line-shaped MP particles appeared reticulated, uneven, flat, and striped, respectively (Fig. 5). In addition, there were different degrees of mechanical damage textures on the particle surfaces, such as that at position a indicated by arrows. We found spindle-shaped micro-organisms (b arrows) on the surface of foam, fragment, sheet, and line-shaped MPs (Fig. 5).

The surfaces of the four shapes of MP particles comprised the elements C, O, S, N, Fe, Mg, Al, Si, and Cl according to SEM-EDS. The surfaces of foam MPs comprised Mg, Al, Si, O, and C, and the surfaces of fragment MPs comprised Cl, S, Fe, O, and C. The surfaces of sheet MPs comprised Cl, S, N, O, and C, and the surfaces of line-shaped MPs comprised S, Si, Al, O, and C. Moreover, oxygen was clustered on surface depressions of and bulges of foam, fragment, sheet, and line MPs (see position c in Fig. 5).

## Discussion

Microplastics are widespread in freshwater environments and pose severe threats to freshwater ecosystem health. Microplastic pollution in inland waters of Asia, especially in China, is more severe than in other parts of the world (Chen et al. 2021). There have been a few reports on MP pollution in the TGR in recent years, showing an increasing trend in MP pollution with abundances of MPs of 1.36×10^7^ items km^−2^ in surface water of the mainstream of the TGR, 1.19×10^7^ items km^−2^ in Xiangxi River in 2015 (Zhang et al. 2015), 3.42×10^7^ items km^−2^ in backwater areas of Xiangxi River in 2017 (Zhang et al. 2017), and 4.70±2.82×10^3^ n m^−3^ in the mainstream of the TGR in 2018 (Di and Wang 2018). In our study, the abundances of MPs reached 5.27±3.50×10^7^ items km^−2^ in the Wanzhou section of the TGR during the flood period, which was higher than previously reported abundances. Accordingly, MP pollution appears to be more severe at some sites of the TGR, especially in the flood period.

The abundance and distribution of MPs are spatially variable in freshwater environments (Treilles et al. 2022). The abundances of MPs were higher in backwater areas near Wanzhou city than in mainstream and tributary areas of the Wanzhou section (Fig. 2), especially in the flood period at the sampling site W8 (Table 1). Previous reports have also shown that the contamination of MPs was more severe in urban areas than in other areas in the TGR (Di and Wang 2018). This may be related to human activities and inputs of urban sewage and surface runoff. Human activities have increased the potential MP pollution risk to the urban rivers and lakes (Wang et al. 2017; Yin et al. 2019), and backwater areas accumulate more MPs than mainstream areas due to slow-flowing water (Zhang et al. 2017). We also found that MP pollution was more severe in the flood period than in the impoundment period, especially in the backwater area. Surface runoff after heavy rain is one of the reasons why MP abundances are higher in the flood period than in the impounding period (Horton et al. 2017). After heavy rain, surface runoff transports MPs from the land and riverbanks into the reservoir, increasing MPs (Scircle et al. 2020). At the same time, slow-flowing water in the backwater areas of the TGR causes high MP retention. In previous studies, we found that the surface water in backwater areas of the TGR is also favorable to organisms (Xiao et al. 2018a; Xiao et al. 2018b; Deng et al. 2021).

In the Wanzhou section of the TGR, the proportion of MPs with a particle size of 1–5 mm in the mainstream, tributaries, and backwater areas was higher in the flood period than in the impoundment period (Fig. 3A). Hydrodynamic conditions (e.g., runoff) in the flood period may have transported plastic particles from land pollution sources into the reservoir, resulting in a high proportion of large size MPs (Treilles et al. 2022). In the impoundment period, 0.3–1 mm MP particles had higher abundances and contributed more than 50% in the tributary (Fig. 3A) Point-source inputs along the rivers, such as sewage outlets, became the main pollution sources, contributing smaller MP particles in the impoundment period (Xiang et al. 2012). Sewage MP particles have been reported to be smaller, usually with a size of less than 1 mm (Chen et al. 2020b).

The four shapes (foam, fragment, sheet, and line-shaped particles) of MPs detected in our study were consistent with the classification of MPs in the waters around the TGD and the Xiangxi River (Zhang et al. 2015; Zhang et al. 2017). However, we found that foam MPs were more abundant than other shapes in the flood period, and fragments were the most abundant shape in the tributaries in the impoundment period (Fig. 3B). Foam MPs were more than other shapes in the 1–5 mm class. However, fragment MPs were more abundant than other shapes in the 0.3–1 mm class (Fig. 4). Shapes of MPs were related to pollution sources and their degradation. Foam particles are mainly derived from commodity transportation and food packaging waste (Liu et al. 2021). Fragment, sheet, and line-shaped particles might come from daily necessity products, such as toiletries, wastewater from household washing machines, fishing appliances, and domestic sewage (Praveena et al. 2021). In addition, PS MP particles were mainly foam-shaped, PE particles were fragment, sheet, and line-shaped, and PP particles were fragment-shaped according to ATR-FTIR. The hydrodynamic conditions of the TGR are weak (Zhang et al. 2015), and the particle density of foamed PS MPs is lower than that of PE and PP MPs. Thus, foamed PS MPs are more prone to transport and occur more frequently in surface water. According to our results, more foam MPs were transported into the TGR with surface runoff in the flood period, while more fragment MPs were discharged into the TGR via sewage in the impoundment period.

Microplastic particles with different shapes had unique surface structural properties in the Wanzhou section of the TGR. There were cracks, pits, and grooves on the surfaces of the four kinds of MP shapes (Fig. 5). Transport by water and wind for long times may cause different degrees of mechanical erosion damage to MP particles (Miskolczi and Bartha 2008). The surfaces of MPs contained O and/or other elements (Mg, Al, Si, Cl, S, Fe, and N) besides C according to SEM energy spectra (Fig. 5). Due to long-term aging, absorption, adhesion, oxidation, or hydrolysis (De Frond et al. 2021; Lee et al. 2021), MPs are decomposed into smaller sizes, and MPs in the reservoir were severely oxidized into PS, PE, and PP particles, and some micro-organisms adhered to the surface depressions of the MPs (Fig. 5, b arrows). The surface characteristics of MPs in the water could facilitate bacterial adhesion and absorption of ions and organic compounds, which should be studied in the future.

## Conclusions

This study is the first to explore seasonal pollution and surface characteristics of microplastics in different habitats (mainstream, tributary, and backwater areas) of an urban section of the TGR in the flood and impoundment periods. Microplastic pollution was the most serious in backwater areas and the flood period compared to other areas and the impoundment period. Pollution with 1–5 mm and foam MP particles was dominant in the Wanzhou section of the TGR. However, in the tributaries, the proportions of 0.3–1 mm and fragment MPs were higher in the impoundment period. There were at least polystyrene (foam), polypropylene (fragment), and polyethylene (fragment, sheet, and line-shaped) MPs present in the Wanzhou section. MPs appeared to originate mainly from surface runoff in the flood period and sewage discharge in the impoundment period. The surfaces of MPs showed damage and oxidation, and different elements were found, warranting further studies of surface characteristics. The results of the present study contribute to our understanding seasonal pollution and surface characteristics of MPs in the Wanzhou section of the TGR and similar watersheds.

## Data Availability

All data generated or analyzed during this study are included in this published article.
